# Nicotinamide Adenine Dinucleotide (NAD)-Dependent Protein Deacetylase, Sirtuin, as a Biomarker of Healthy Life Expectancy: A Mini-Review

**DOI:** 10.2174/0118746098319674240827104612

**Published:** 2024-10-04

**Authors:** Yodo Sugishita, Yuki Suzuki-Takahashi, Kazuo Yudoh

**Affiliations:** 1 Department of Frontier Medicine, Institute of Medical Science, St. Marianna University School of Medicine, Kawasaki City, Japan

**Keywords:** Aging, healthy life expectancy biomarkers, surrogate markers, sirtuin, nicotinamide adenine dinucleotide (NAD), mitochondria

## Abstract

Although a variety of disease-specific biomarkers have been identified for common lifestyle- or aging-related diseases, there are currently no indices available to measure general health or the existence of pre-symptomatic conditions in various types of tissue and organ damage. A rising body of research suggests that sirtuins may have the potential to be used as an index to assess overall health status and the existence of pre-symptomatic illness states. Sirtuins (SIRTs) are nicotinamide adenine dinucleotide (NAD)-dependent deacetylases expressed in a variety of human somatic cells both in health and disease conditions. The activity and expression of SIRTs affect important metabolic pathways, such as cell survival, senescence, proliferation, energy production, stress tolerance, DNA repair, and apoptosis, thereby closely linked to aging and longevity. Given the broad significance of SIRTs in physiological function maintenance, their activity in somatic cells may reflect the early cross-sectional status of tissue damage caused by aging or systemic inflammatory responses that are too early to be detected by disease-specific biomarkers. In this mini-review, we discuss the utility of SIRTs as a surrogate clinical biomarker for health status to evaluate and monitor health life expectancy and the presence of pre-symptomatic illness states.

## 1 INTRODUCTION

Japan is renowned for having one of the world’s most elderly populations, boasting an average life expectancy of 90 years [1]. It is estimated that the population over the age of 65 will account for more than 30% of the total population by 2025 [1]. Concurrently, the bedridden population has increased, resulting in an associated surge in annual medical costs. These costs recently reached 59 trillion JPY (US$ 444 billion) and as high as 94 trillion JPY (US$ 707 billion) when long-term care expenses are considered [1]. In order to reduce the excessive burden on medical institutions and skyrocketing medical costs, it is imperative to prolong people’s healthy life expectancy, which is the period when they can expect to live in full health. The Japan National Institute of Health and Nutrition has reported a 10-year difference between life expectancy and healthy life expectancy [1]. To address the challenges in extending healthy life expectancy, it is important to promote self-care and self-medication and improve the overall health of the population. However, many people do not receive regular health checks. Even in countries with high levels of health consciousness, like Japan, only 53.5% of Japan’s population undergoes yearly health checks [1-3].

The challenging task to promote self-care and self-medication is that currently, there is no indicator that can objectively assess health status during the pre-symptomatic state. Although various disease biomarkers have been identified for cancer, lifestyle- or aging-related diseases, none have yet been identified to measure general health status. Therefore, it is essential to identify a simple and affordable evidence-based biomarker that can accurately measure general health status. Identifying a universal biomarker to measure general health status will be a significant breakthrough in preventive medicine, which can lead to early diagnosis and early intervention to prevent disease and promote health during aging.

Our research has focused on sirtuin (SIRT), a longevity-related deacetylase. There is growing evidence that the level of SIRT protein expression in somatic cells may be able to foretell the cross-sectional state of tissue/organ damage due to cellular deactivation, senescence, or systemic inflammation [4-7]. These early stages of cross-sectional tissue/organ damage cannot be detected by biomarkers specific to the disease. Hence, SIRT expression may be a more comprehensive indicator of systemic health than specific biomarkers for individual diseases.

In this mini-review, we summarize the roles of SIRT in important metabolic pathways, namely, response to cellular stress and regulate cellular metabolism, and its potential to be used as a surrogate biomarker to evaluate and monitor healthy life expectancy.

## 2 LONGEVITY-RELATED DEACETYLASES, SIRTS, AND THEIR ROLES IN CELL BIOLOGY

Research interests in SIRTs have increased steadily after it was reported that overexpression of Sir2 could extend yeast lifespan by as much as 70% [8, 9]. In mammals, the SIRT family consists of seven members (SIRT-1 to -7), among which SIRT-1 is the best-studied family member and shares the highest sequence homology to SIRT-2 in yeast. SIRTs have highly conserved catalytic domains, yet each SIRT has distinct expression patterns, catalytic activities, and ultimately - biological functions. Sirtuin family members show their functions through the mechanism involving the deacetylation of target proteins in the different intracellular localizations (Table **1**). The expression of SIRT-1, SIRT-6, and SIRT-7 are predominantly found in nuclear, SIRT-2 in cytosolic, and SIRT-3, SIRT-4, and SIRT-5 in mitochondria [10]. The SIRTs belong to nicotinamide adenine dinucleotide (NAD)-dependent enzymes that mostly display protein deacetylase activity except for SIRT-4, which is known to catalyze the transfer of an ADP-ribose molecule from NAD+ to a target protein. Modulation of these activities has broad implications in cell biology, such as cell differentiation, cell growth, energy metabolism, stress tolerance, DNA repair, apoptotic programmed cell death, inflammation, antioxidative response, as well as the regulation of cellular aging in a variety of cells [8-13]. Members of the SIRT family (SIRT-1 to -7) act by deacetylating target proteins in different cellular compartments (Table **1**) [8-11, 14, 15].

**Fig. (1):**
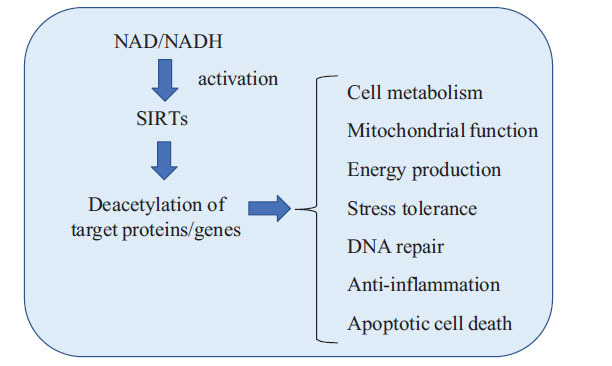
Role of SIRTs, nicotinamide adenine dinucleotide (NAD)-dependent protein deacetylases, in the cell biology: SIRTs are involved in stress responses by regulating the programed cell death and cellular metabolism through the deacetylation of target proteins in human somatic cells. NADH: reduced type of NAD.

Recent studies indicate that SIRT may have two important roles: “response to cellular stresses” and “regulation of cellular metabolism”, which have been implicated in the pathophysiology and pathogenesis of a variety of diseases [16, 17]. SIRTs are involved in stress responses by regulating apoptosis and cell metabolism through the deacetylation of target proteins in normal or malignant tumor cells shown in (Fig. **1**) [7, 12, 13, 16]. Several reports have demonstrated that SIRTs are key regulators of several molecules and genes during both anabolic and catabolic reactions in response to mechanical, inflammatory, and oxidative stress in a number of diseases [6, 7, 9, 12, 13, 16-19]. A better understanding of the involvement of SIRT in “stress tolerance” and “cellular metabolism” could lead to new insights into the development of novel diagnostic tools and therapies for a variety of diseases. In response to varied cellular stresses, SIRT activation may alter both the cellular activity and the energy metabolism of various tissues and organs in the body, thereby heavily influencing health and healthy life expectancy. Thus, monitoring SIRT activity in human somatic cells may predict the cross-sectional state of various tissue/organ damages due to aging, cellular deactivation, senescence, and systemic inflammatory responses that cannot be detected by tissue-specific or disease-specific biomarkers. In addition, SIRT may have the potential to be a biomarker for monitoring health and pre-symptomatic conditions.

### 2.1 Effect of SIRT on Stress Tolerance (Response to Cellular Stresses)

Extrinsic cell stresses, such as mechanical and oxidative stress, inflammation, and aging, have been recognized as major risk factors for several diseases [14, 19-25]. In response to stresses, altered cellular metabolism leads to an imbalance between catabolic and anabolic reactions and facilitates the progression of diseases [25-27]. For example, SIRTs have been shown to promote the expression of chondrocyte differentiation-specific genes and suppress the expression of apoptotic proteins in chondrocytes in articular cartilage tissue [18, 20, 28, 29]. However, in response to catabolic stresses, downregulation of SIRT activity may result in increased chondrocyte apoptosis and suppression of chondrocyte anabolism in articular cartilage tissue, consequently leading to reduced stress tolerance and damaged cartilage tissues.

Mechanical stress is another critical disturbance factor in tissue/organ homeostasis [30-32]. Recently, it has been suggested that members of the SIRT family, particularly SIRT-1, can regulate cellular responses against mechanical stress [6, 7, 13, 22, 33, 34] as well as tolerance to other stresses [35-37]. In the vascular system, mechanical shear stress caused by blood flow is known to be essential for maintaining vascular homeostasis [38-40]. The continuous exposure of vascular smooth muscle cells to blood flow stress regulates and maintains vascular endothelial cell activity and vascular tone, which are controlled by mechanisms involving the SIRT pathway [38, 40]. In addition to vascular vessels, it has been demonstrated that mechanical stress enhances the expression of genes encoding proinflammatory cytokines and chemokines, as well as their receptors, *via* the activation of SIRT in periodontal ligament cells, suggesting that mechanical force activates periodontal ligament cells to accelerate immune reactions *via* SIRT activation [41]. In bone biology, the SIRT pathway may also function as a key mediator of osteoblastic bone formation in response to mechanical stress. However, the trigger mechanism that modulates the SIRT pathway following mechanical stress-induced osteoblast differentiation and bone formation remains unknown [33, 42].

### 2.2 Effect of SIRT on Cellular Metabolism

In addition to the role of SIRT-1 as a key regulator of stress tolerance, several studies have indicated that SIRT plays an important role in cellular metabolism, particularly glucose metabolism (energy production) [8, 9, 26, 43, 44]. There is a consensus that adenosine monophosphate-activated protein kinase (AMPK) also regulates cellular energy metabolism [43-45]. Once activated, AMPK responds by phosphorylating downstream target proteins, thereby activating pathways that produce adenosine triphosphate (ATP) [45, 46].

**Fig. (2):**
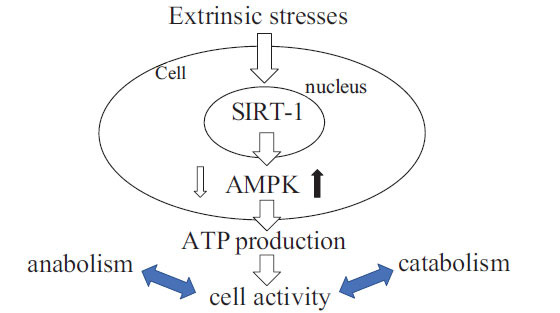
SIRT-1 and adenosine monophosphate-activated protein kinase (AMPK) as an energy sensor in cell metabolism: SIRT-1 regulates energy metabolism (ATP production) through the modulation of AMPK activity in human somatic cells. The extrinsic stresses may regulate the SIRT-1 activity and its target AMPK, facilitating the decrease of cellular energy (ATP) and cellular activity.

**Fig. (3):**
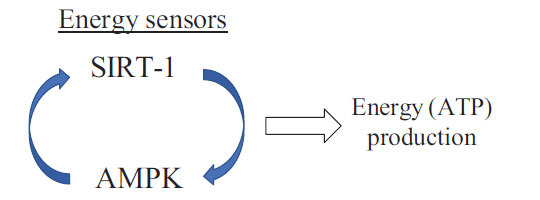
SIRT–AMPK positive feedback in the energy metabolism: SIRT-1 may regulate cellular energy metabolism through the SIRT1-AMPK energy sensor loop.

Interestingly, SIRT-1 inactivation leads to a decrease in both AMPK activity and the production of ATP in somatic human cells, suggesting that it mediates the activation of AMPK and the resulting energy (ATP) production during energy metabolism (7,33). These findings suggest that SIRT-1 may mediate ATP production through the activation of another energy sensor, AMPK, during energy metabolism in somatic human cells shown in (Fig. **2**). In contrast, several studies reported that activation of AMPK increases SIRT-1 activity in a variety of cell types, suggesting the existence of a positive feedback loop between SIRT-1 and AMPK in somatic human cells [47-49] shown in (Fig. **3**). SIRT-1 and AMPK are thus now recognized as critical energy sensors that can regulate the cellular energy balance. Although further studies are needed to clarify the mechanism of the SIRT-AMPK feedback loop in energy metabolism, we speculate that the SIRT–AMPK feedback loop may modulate the AMPK-ATP energy metabolic pathway, and that interfering with this feedback loop might affect cellular energy balance and homeostasis. In addition, disruption of the SIRT*–*AMPK feedback loop may not only disrupt cellular energy metabolism but also decrease cellular stress resistance (stress tolerance) in a variety of cells [7, 42, 47-50]. Next, we discuss the interaction between cellular energy metabo-lism and stress tolerance from the point of view of SIRT biology.

### 2.3 Energy Metabolism is Affected by the Stress Response *via* the SIRT-AMPK System

As mentioned above, SIRT plays critical roles in regulating energy metabolism and in controlling responses to cellular stresses through the regulation of target proteins, and it is closely linked to the pathophysiology of various stress-induced diseases [6, 7, 12, 13, 16-19, 43, 44]. Cellular stresses, such as mechanical and oxidative stress, inflammation, and senescence, may affect cellular energy metabolism *via* a mechanism regulated by SIRT-1 and AMPK [7, 9, 12, 13, 16-19, 26, 43, 44] shown in (Figs. **2** and **3**).

Indeed, it has been demonstrated that cellular stresses, such as nutrient deprivation, hypoxia, mechanical stress (exercise), inflammation, and aging, affect the activation of intracellular AMPK [7, 48-50]. Our previous studies revealed that extrinsic stresses affect the activity of both SIRT-1 and AMPK in articular chondrocytes, consequently facilitating the downregulation of cellular energy metabolism and cartilage matrix homeostasis [7]. Our findings indicate that inflammatory stress, such as the expression of proinflammatory cytokines, inhibits the expression of SIRT-1 in chondrocytes and that SIRT-1 insufficiency enhances the stress-induced decrease of ATP production by chondrocytes [7]. These data also suggest that the activity of the energy sensors SIRTs and AMPK and their regulated energy metabolism are closely linked to cellular stress responses, including mechanical stress, oxidative stress, and inflammation [7-9].

Given the change in cellular energy metabolism during the progression of the disease, we speculate that the build-up of disease-related catabolic stresses may disrupt cellular energy metabolism *via* a change in SIRT activity and the pathways it regulates, which then affects cellular activity, tissue/organ homeostasis and subsequently predisposes the whole body to damage or to senescence [4, 5, 51, 52]. Disturbances in the maintenance of energy metabolism involving the SIRT-APMK pathway may result in the downregulation of cellular activities in the whole body, facilitating a reduction in systemic health, the development of sickness, and/or senescence.

## 3 SIRT AS A BIOMARKER MONITORING HEALTH CONDITIONS AND AGING

Members of the SIRT family act non-redundantly by deacetylating target proteins in different intracellular localizations (Table **1**) [8-11]. SIRT-2, which is mainly cytoplasmic, is closely involved in stress responses by regulating the cell cycle, mitotic checkpoint, programmed cell death, and apoptosis through the deacetylation of target proteins (Table **1**) [9, 11]. Recent studies have shown that SIRT-2 decreases the level of reactive oxygen free radicals (oxidative stress) in cells and deacetylates transcription factors in response to caloric restriction [51-54], suggesting that SIRT-2 plays a role in metabolic regulation, caloric restriction, and anti-oxidative activity, which are closely implicated in the regulation of aging.

Indeed, exercise is thought to accelerate the activities of SIRT-1, -SIRT-2, and SIRT-3 in several tissues [51, 52, 55, 56]. Appropriate exercise may promote health and anti-aging factors *via* SIRT activation since SIRTs influence cellular energy metabolism and stress tolerance, as mentioned above. North *et al.* demonstrated that SIRT-2 induces the expression of the mitotic checkpoint kinase BubR1, which is a key regulator of the spindle assembly checkpoint and has emerged as a key regulator of aging and longevity in mice [51]. Their findings clearly indicate that the activation of SIRT-2 opposes aging and age-related diseases. Furthermore, Kumar *et al.* have reported that lower SIRT-1, SIRT-2, and SIRT-3 levels were significantly associated with frailty in older individuals [52]. They concluded that the level of SIRT activity may play a role as a distinctive biomarker for frailty in elderly people.

**Fig. (4):**
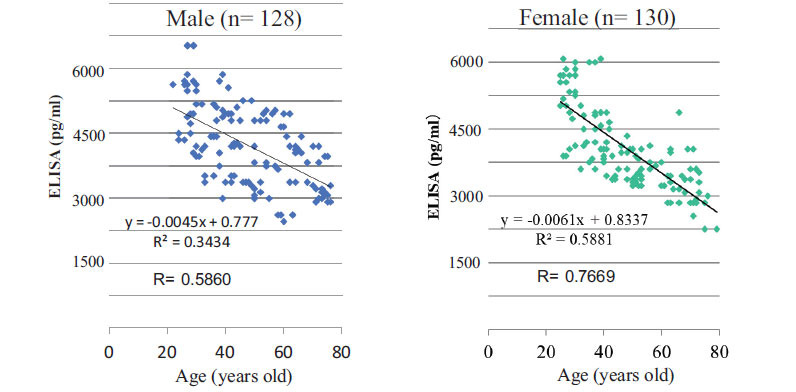
Serum concentration of SIRT-2 in PBMCs. (partial modification of figures in Current Aging Res., 2015): The healthy control group consisted of 258 volunteers (aged 26 to 77 years old, male: 128 cases, female: 130 cases). The protein level of SIRT-2 in PBMCs was significantly decreased with donor age. An inverse correlation between the SIRT-2 level and donor age was observed (*P*<0.01).

Sirtuins are intracellular proteins but not secretions from cells (Table **1**). As sirtuins are not secreted proteins from the cells in the blood, to measure the concentration of sirtuins in PBMCs, the nuclear fractions for SIRT-1, SIRT-6, and SIRT-7, the mitochondrial fractions for SIRT-3, SIRT-4, and SIRT-5 are required to extract from intracellular proteins. In contrast, the SIRT-2 protein exists rich in the cytoplasm. Therefore, extraction processes of intracellular organs are not needed for the sirtuin-2 measurement. The concentration of SIRT-2 may be easily measured in comparison with other SIRTs. As a surrogate biomarker for the presence of pre-symptomatic conditions and healthy life expectancy, it is required to easily measure the biomarker. We demonstrated that lower levels of SIRT-2 in human peripheral blood mononuclear cells correlated with the advance of donor age in both men and women (Fig. **4**) [4, 5]. Our previous study revealed that SIRT levels are lower in older people than in younger people.

Recently, there has been increasing evidence showing that SIRT expression induces aging, including SIRT-7 [55-57] as well as SIRT-1, SIRT-2, and SIRT-3 [51, 52, 58-60]. In a recent review, it has been demonstrated that the SIRT-6 level in the nucleus of cardiomyocytes is associated with global health through the control of cardiovascular function [61]. SIRT-6 in the nucleus may regulate the mitochondria function in cardiomyocytes. In addition, it has been clearly indicated that SIRT-1 in the nucleus maintains a mitochondrial function [62]. As mitochondrial dysfunction associated with mitochondrial DNA mutations, enzyme defects, generation of ROS, and altered oxidative homeostasis is well known to induce carcinogenesis, health conditions, and aging, the SIRT-1 activity in the nucleus is thought to participate in global health through the maintenance of mitochondrial function [62]. Recently, it has also been reviewed that mitochondria-relevant nutraceuticals may have the potential to maintain health conditions and to prevent the disease condition through the activation of mitochondrial sirtuins, which are associated with metabolism, senescence, and longevity [61, 62]. Although further studies are needed to clarify whether SIRT activity is induced by aging and whether its activity is altered through medical conditions, medicine, foods, supplements, smoking, or physical exercise, the SIRT level in PBMCs may have potential as a biomarker to monitor health and aging that are widely and transversely influenced by inflammation, cellular senescence, or degeneration of organs and tissues as a result of a change in the cellular activity.

## 4 PRACTICAL APPLICATIONS OF SIRT AS A BIOMARKER

With the establishment of SIRT-2 as a surrogate biomarker for the presence of pre-symptomatic conditions and healthy life expectancy, we expect the following opportunities, mainly in the areas of self-care and self-medication:

### 4.1 Practical Application of SIRT Assessment During Medical Examinations (SIRT-2 as an Index for Health Management and Goal Setting)

We believe that by measuring SIRT-2 levels in the blood and comparing them to median SIRT-2 expression levels in the same age group or by monitoring SIRT-2 expression levels over time, we can use this as an index for health management and assessment, as well as for improving personal health care and self-medication.

### 4.2 Practical Application of SIRT as an Index for the Efficacy of Health Food Products, Drugs, and Supplements and for R&D Screening

SIRT-2 is used as an index to determine the effectiveness of foods, pharmaceuticals, supplements, *etc*. For the purpose of health maintenance, it can be persuasive and help boost their sales. In the development of new potential substances, it can also be applied to screen their efficacy on health improvement and contribute to the development of health-related products by providing scientific evidence. In addition, the following age-related changes in tissues and organs can be easily monitored by combining tissue-specific or disease-specific biomarkers with SIRT as a surrogate biomarker and new business developments for self-health care can be anticipated.

## CONCLUSION

To maintain a stable social infrastructure and to create a society in which everyone can live with peace of mind, it is essential to promote healthy life expectancy, self-care, and self-medication, not only for the elderly but also for middle-aged and young adults. Therefore, it is necessary to know our own comprehensive health status.

Based on the SIRT biology mentioned above, measuring the expression of SIRT proteins, particularly SIRT-1,-2,-3, and -7, in somatic cells may allow us to monitor tissue/organ damage owing to aging, senescence, and inflammation across tissues and organs, which cannot be detected by biomarkers specific to those tissues/organs or diseases. SIRT may serve as a biomarker to monitor health and evaluate healthy life expectancy. This is expected to have the following social and economic ripple effects:

- It enables an accurate measurement of one’s health and could be used as a motivator to improve lifestyle.
- By promoting self-management of one’s health, it will enable self-observation of pre-symptomatic conditions and overall health. Lastly, it can promote the extension of healthy life expectancy for consumers and reduce medical costs through prevention and early detection of diseases.

## Figures and Tables

**Table 1:** Sirtuin (SIRT) family.

**SIRTs**	**Target Proteins**	**Function**	**Localization**
SIRT-1	NF-kB, p53 FOXO, PGC-1a	Stress tolerance Cell metabolism	Nucleus Cytoplasm
SIRT-2	Tubulin, FOXO	Cell cycle, Apoptosis Cell differentiation	Cytoplasm
SIRT-3	GDF, ACS2	Themogenesis ATP production	Mitochondria
SIRT-4	GDH, IDE, ANT	Insulin secretion	Mitochondria
SIRT-5	CPSI	Urea circuit	Mitochondria
SIRT-6	NF-kB, Histon H3	Cell metabolism Base excision repair	Nucleus
SIRT-7	RNA polymerase 1	rDNA tasnsfer	Nucleus

**Abbreviations:** LNF-kB: nuclear factor-kB, Foxo: forkhead box O, PGc-1a: peroxisome proliferator-activated co- activater 1a, GDF: glutamate dehydrogenase, ACS2: acetyl-CoA synthetase 2, IDE: insulin-degrading enzyme, ANT: adenine nucleotide translocase, CPSI: carbamoyl phosphatase synthetase 1.

## LIST OF ABBREVIATIONS

AMPK: Adenosine Monophosphate-activated Protein Kinase
ATP: Adenosine Triphosphate
NAD: Nicotinamide Adenine Dinucleotide
SIRTs: Sirtuins
